# The Association Between Prolactin and Metabolic Parameters in PCOS Women: A Retrospective Analysis

**DOI:** 10.3389/fendo.2020.00263

**Published:** 2020-05-12

**Authors:** Haiyan Yang, Junbo Di, Jiexue Pan, Rong Yu, Yili Teng, Zhuhua Cai, Xiaohui Deng

**Affiliations:** ^1^Center for Reproductive Medicine, Department of Obstetrics and Gynecology, Qilu Hospital, Cheeloo College of Medicine, Shandong University, Jinan, China; ^2^Reproductive Medicine Center of the First Affiliated Hospital, Wenzhou Medical University, Wenzhou, China; ^3^Qilu Children's Hospital, Cheeloo College of Medicine, Shandong University, Jinan, China

**Keywords:** PRL, polycystic ovary syndrome, metabolism, IVF-ET, infertility

## Abstract

**Background:** The aim of this retrospective study was to analyze the association between prolactin (PRL) and metabolic parameters in infertile patients with polycystic ovary syndrome (PCOS).

**Methods:** A total of 2,052 patients with PCOS and 9,696 patients with tubal infertility (non-PCOS) undergoing *in vitro* fertilization and embryo transfer (IVF-ET) at the reproductive medicine center of the first affiliated hospital of Wenzhou Medical University from January 2007 to July 2017 were enrolled in this study. Serum PRL, basic endocrine hormones, fasting plasma lipid, fasting plasma glucose (FPG), liver function, thyroid hormone and other parameters were measured and analyzed.

**Result:** PRL levels were significantly lower in PCOS patients than controls over all age groups (*p* < 0.05). In the PCOS patients, serum PRL was significantly and positively correlated with FPG, serum TSH and serum FT4, and significantly and negatively correlated with LH, LH/FSH, TC, TG, LDL-C, AST, ALT, γ-GGT, FT3, and FT3/FT4 (*p* < 0.05 or 0.01). After adjusted for age and body mass index (BMI), serum PRL was positively correlated with FPG, TSH, and FT4, and negatively correlated with LH and LH/FSH.

**Conclusion:** Low serum PRL may be an important cause of metabolic risk in infertile patients with PCOS.

## Background

Studies have demonstrated that prolactin (PRL) has more than 300 other functions in addition to regulating the development of breast and lactation (1–5). Among them, the regulatory roles of PRL in metabolism has been a focus of research in recent years.

PRL may be secreted by macrophages in the adipose tissue in response to inflammation and hyperglycemia (6), promoting the formation of adipose and inhibiting the decomposition of lipid in the adipose tissue (7). The role of PRL in promoting insulin secretion in islets is well-demonstrated (8). PRL stimulates the proliferation and survival of islet β cells, improves the quality of β cells and enhances the secretion of insulin and sensitizes the liver to insulin (9–11). Study has shown that PRL can affect metabolism homeostasis, glucose and lipid storage by regulating key enzymes and transporters related to glucose and lipid metabolism in the target organs (12). Therefore, PRL has been suggested to be closely associated with insulin resistance, hypertension, thrombembolia, stroke and coronary syndrome (13–17).

Serum PRL is closely related to metabolism. It has been demonstrated that there is a significant association between hyperprolactinemia and all-cause mortality and specific cardiovascular disease mortality (18, 19). However, the associations between PRL and metabolic risk factors are different when serum PRL is within and outside the normal range. Low serum PRL within the physiological range has a negative correlation with insulin sensitivity and plasma glucose level (20–22), and is often associated with poor metabolic outcomes of metabolic syndrome and type 2 diabetes (23–25). Muldon et al. confirmed that person meeting either the National Cholesterol Education Program or International Diabetes Federation criteria for the metabolic syndrome has lower mean PRL response (26). Ruiz-Herrera et al. and a large cohort study in 2013 also confirmed that lower level of serum PRL disrupts the metabolic balance (21, 27). PRL levels in men and women with impaired glucose tolerance, type 2 diabetes and insulin resistance and children with metabolic syndrome and obesity are lower, and might increase after lifestyle intervention in obese children (23). In other words, the high serum PRL within normal physiological range gives rise to better insulin sensitivity, better distribution of adipose tissue and ultimately improved metabolic disorders. However, if the level is outside the normal physiological range, the effect would reverse (19).

Despite of numerous studies, little is known about the correlation between serum PRL and metabolism in infertile females. PCOS patients are a very important subset of infertile women. PCOS is closely associated with hyper-inflammation, insulin resistance, impaired glucose tolerance, metabolic syndrome, hyperlipidemia, hypertension, and increased incidence of cardiovascular disease (28–33). Glintborg et al. found that serum PRL in hirsutism and/or PCOS patients are significantly lower than in healthy controls, and are negatively correlated with LDL-C (34). This study aimed to analyze the serum PRL level in a large number of infertile PCOS patients and its correlation with various types of metabolic indicators to better understand the relationship between PRL and metabolism.

## Methods

### Patients

This was a retrospective study including a total of 2,052 PCOS patients and 9,696 patients with tubal infertility (non-PCOS) who underwent *in vitro* fertilization and embryo transfer (IVF-ET) from January 2007 to July 2017 at the reproductive medicine center of the first affiliated hospital of Wenzhou Medical University. PCOS patients were enrolled according to the diagnostic criteria recommended by ESHRE/ASRM in 2004 (35). All patients were Chinese women and were included if they were treated with IVF-ET (fresh cycle) for the first time and had the normal range of serum PRL levels. Because the legal marriage age is 20 years and up and only legally married women are eligible for IVF-ET, and the incidence of metabolic disorders such as endocrine dysfunction, hypertension, hyperlipidemia and diabetes will increase after 40 years old, the inclusive age of the patients in the study was set at 20–40 years to minimize the impact of other healthy conditions of patients on the results of the study.

Patients were excluded if she smoked, treated with any medication in 3 months, had hypertension, diabetes, hyperlipemia, cardiovascular disease, hyperthyreosis, or hypothyroidism, positive HBsAg reaction, C hepatitis, abnormal liver function, chronic nephritis, renal dysfunction or pituitary microadenoma. Patients were also excluded if she had unexplained infertility, recurrent abortion, congenital abnormalities, congenital adrenal hyperplasia, Cushing syndrome or androgen secreting tumors. Informed consent was obtained from every patient and the study protocols were approved by the Ethics Committee of the First Affiliated Hospital of Wenzhou Medical University.

### Sampling and Patient Data Collection

Patient information about smoking, diseases, past-operation and medical therapy were questioned and recorded. Fasting blood samples were collected between 9 and 11 am in the morning at least 2 h after wake-up and 8 h after fasting on day 2–5 of a menstrual cycle. The height (m) and weight (kg) were measured to calculate body mass index [BMI, BMI = weight (kg)/height (m^2^)]. Blood pressure was taken twice in an interval of 2 min after at least 10 min rest using a mercury sphygmomanometer.

### Laboratory Tests

PRL, FSH, LH, T, E_2_ TSH, FT3, and FT4 levels in blood samples were measured using chemiluminescence assay on UniCel® DxI 800 Immunoassay System (Beckman Coulter, USA) with commercial kits (Access Prolactin, Access hFSH, Access hLH, Access Testosterone, Access Estradiol, Access TSH, Access Free T3 and Access Free T4, Beckman Coulter, USA) according to manufacturer's and supplier's instructions. FPG, TC, TG, LDL-C, HDL-C, uric acid and liver function were measured using Cobas 8000 modular analyzer and kits [Glucose HK, Gen.3 (GLUC3), Triglycerides (TRIGL), Cholesterol Gen.2 (CHOL2), Aspartate Aminotransferase (ASTL), Alanine aminotransferase acc. to IFCC (ALTL), r-Glutamyltransferase ver. 2(GGT-2) and Alkaline Phosphatase acc. to IFCC Gen.2 (ALP2), Diagnostics Gmb, Germany; Cholestest LDL, Cholestest HDL, SEKISUI MEDICAL CO., LTD. Japan] according to manufacturer's instructions. The detection limit for PRL was 20 mIu/L. If serum PRL is over >25 μg/L (or 530 mIu/L), it is considered to be hyperprolactinemia (36, 37). However, due to variation in detection method and kit used, the normal range of PRL varies slightly among hospitals. Based on the method and kit used at our hospital and published consensus on diagnosis and treatment of hyperprolactinemia, the normal range was set between 70.81 and 566.46 mIu/L (38).

### Statistical Analysis

The normal distribution of continuous data was tested by Shapiro-Wilk test. Parameters were not normally distributed and were therefore described using medians and quartiles. The Mann-Whitney test was used to compare differences between PCOS patients and controls. The Kruskal-Wallis test was used to compare differences among the four PCOS patient groups according to PRL level. The Chi-square test was used to compare the differences in frequencies between groups. Regression analyses were used to adjust for differences in BMI between PCOS patients and controls. The correlation among variables was analyzed using the Spearman correlation analysis. Multivariate linear regression was used to analyze the effect of variables on PRL. All statistical analyses were performed with SPSS 22. *p* < 0.05 were considered significant.

## Results

### Effect of Age-Groups on PRL, Endocrine, and Other Metabolic Parameters

After adjusted for BMI, the levels of PRL and FSH were significantly lower, while systolic blood pressure (SBP), diastolic blood pressure (DBP), LH, LH/FSH, testosterone (T), TG, TC, LDL-C, AST, ALT, γ -GGT, ALP, and uric acid were significantly higher in PCOS patients than in non- PCOS patients group (Table 1; *p* < 0.05 or *p* < 0.01) in all age groups. PCOS patients older than 31 years had significantly lower HDL-C level and significantly higher FT3/FT4 than control (Table 1; *p* < 0.05 or *p* < 0.01 and Supplementary Table 1).

**Table 1:** Comparison of PRL, endocrine, and other metabolic parameters in PCOS and controls of different age groups.

**Age (years)**								
	**≤25**		**26–30**		**31–35**		**36–40**	
	Non-PCOS (*n* = 887)	PCOS (*n* = 258)	Non-PCOS (*n* = 3,786)	PCOS (*n* = 1,146)	Non-PCOS (*n* = 3,352)	PCOS (*n* = 543)	Non-PCOS (*n* = 1,671)	PCOS (*n* = 105)
Age (year)	24 (23–25)	24.5 (24–25)^*^a	28 (27–29)	28 (27–29)^**^b	33 (32–34)	32 (31–34)^**^b	37 (36–39)	37 (36–37)^**^b
Systolic blood pressure (mmHg)	112 (105–120)	116 (105–123)^*^	112 (103–120)	116 (106–125)^**^b	112 (104–121)	118 (107–126)^**^a	115 (105–124)	120 (109–128)^*^a
Diastolic blood pressure (mmHg)	70 (66–76)	71 (68–78)^*^a	70 (65–76)	72 (68–80)^**^b	70 (66–77)	73 (69–80)^**^	72 (67–79)	75 (70–82)^**^a
BMI(kg/m^2^)	20.40 (18.90–22.38)	22.09 (19.44–24.73)^**^	20.70 (19.20–22.70)	22.40 (20.20–25.40)^**^	21.23 (19.63–23.15)	23.44 (20.89–25.81)^**^	22.03 (20.31–24.0)	23.73 (22.0–25.44)^**^
PRL (mIU/L)	289.63 (222.98–385.40)	272.01 (205.18–364.62)^*^a	295.09 (224.70–380.32)	256.11 (199.87–338.46)^**^b	270.41 (202.88–358.40)	239.39 (183.85–334.24)^**^a	249.69 (188.35–336.82)	222.41 (191.66–290.01)^*^a
LH (IU/L)	3.35 (2.10–4.51)	9.68 (5.32–10.18)^**^b	4.49 (3.43–5.96)	8.31 (5.27–12.90)^**^b	4.36 (3.26–5.76)	8.05 (4.80–12.01)^**^b	3.22 (2.77–4.70)	6.60 (4.82–11.56)^**^b
FSH (IU/L)	7.39 (6.22–8.27)	6.40 (6.37–7.97)^**^b	7.47 (6.35–8.79)	6.68 (5.67–7.79)^**^b	7.68 (6.52–9.07)	6.68 (5.70–7.75)^**^b	7.97 (6.70–9.74)	6.51 (5.65–7.36)^**^b
LH/FSH	0.61 (0.47–0.81)	1.27 (0.88–2.0)^**^b	0.60 (0.45–0.80)	1.26 (0°82–1.90)^**^b	0.55 (0.41–0.74)	1.19 (0.76–1.80)^**^b	0.49 (0.36–0.65)	1.13 (0.73–1.45)^**^b
E_2_ (pmol/L)	159.5 (95–200.5)	95.4 (87–121)	137 (98–187)	145 (104–195)^*^b	141 (102–195)	146 (106–198)^*^a	130 (73–158)	215 (195–326)
T (nmol/L)	1.36 (0.88–1.53)	1.49 (1.44–1.75)^**^b	1.30 (1.00–1.66)	1.82 (1.41–2.32)^**^b	1.21 (0.95–1.55)	1.79 (1.37–2.28)^**^b	1.14 (0.88–1.40)	1.90 (1.19–2.59)^**^b
FPG (mmol/L)	5.2 (5.0–5.6)	5.2 (5.0–5.5)	5.3 (5.0–5.8)	5.2 (5.0–5.6)	5.0 (4.9–5.3)	5.2 (5.1–5.4)	5.75 (5.1–5.7)	5.6 (4.7–5.7)
TG (mmol/L)	0.83 (0.61–1.15)	1.09 (0.78–1.55)^**^a	0.85 (0.63–1.21)	1.13 (0.76–1.75)^**^b	0.89 (0.65–1.25)	1.26 (0.87–1.93)^**^b	0.95 (0.70–1.35)	1.49 (0.98–2.03)^**^b
TC (mmol/L)	4.21 (3.76–4.68)	4.48 (3.99–5.12)^**^b	4.3 (3.84–4.84)	4.56 (4.04–5.15)^**^b	4.43 (3.93–4.94)	4.69 (4.23–5.25)^**^b	4.55 (4.09–5.08)	4.93 (4.39–5.47)^**^b
LDL-C (mmol/L)	2.21 (1.66–2.52)	3.06 (1.70–3.22)^**^b	2.35 (1.98–2.79)	2.56 (2.14–3.08)^**^b	2.43 (2.06–2.86)	2.67 (2.25–3.13)^**^b	2.52 (2.14–2.95)	2.86 (2.42–3.30)^**^b
HDL-C (mmol/L)	2.28 (2.53–3.09)	1.37 (1.16–1.61)^*^	1.44 (1.24–1.66)	1.34 (1.15–1.58)^*^	1.43 (1.23–1.64)	1.31 (1.13–1.57)^**^a	1.43 (1.23–1.64)	1.27 (1.11–1.46)^**^a
AST (U/L)	18 (15–20)	18 (16–23)^*^a	18 (15–21)	19 (16–23)^**^b	17 (15–21)	19 (16–24)^**^a	19 (15–23)	22.5 (20–28)^**^a
ALT (U/L)	13 (10–18.5)	19 (15–29.5)^**^a	12 (10–19)	17 (13–24.5)^**^b	14 (11–19.5)	21.5 (14–31)^**^b	15 (12–19.5)	25 (18–42)^**^a
γ -GGT (U/L)	12 (10–16)	15 (12–19)^**^a	13 (11–16)	16 (12–25)^**^b	13 (11–17)	18 (13–29)^**^b	16 (11–26)	17.5 (14–32)^**^b
ALP (U/L)	59 (52–67.5)	66 (61.5–78)^**^b	58 (50–68)	64 (57–78)^**^b	57 (50–66.5)	66 (54–77)^**^b	59 (49–72.5)	65 (51–74)^**^a
TBIL (umol/L)	11.5 (9.5–14)	12 (9.5–14)^*^	12 (9–14)	11 (9–13)^**^	12 (10–15)	11 (9–14)^*^	13 (10.5–15)	14 (14–17)^*^
DBIL (umol/L)	3 (2–4)	3 (2–3.5)	3 (2–4)	3 (2–3.5)	2 (3–4)	3 (2–3)	3 (3–4)	4 (3–4)
TP (g/L)	76.7 (73.9–78.9)	78.0 (75.4–80.6)	75.9 (73.6–78.6)	76 (73.4–80.0)^**^a	76.1 (73.7–79)	77.3 (74.3–79)^*^a	75.8 (73.05–78.3)	76.3 (74.2–77.4)
ALB (g/L)	47.2 (45.6–48.6)	48.2 (43.9–50.2)	47.2 (45.4–49.0)	47.3 (45.5–49.2)	47.1 (45.1–48.9)	47.5 (45.8–48.9)	46.9 (44.8–48.1)	46.8 (46–47.1)
GLB (g/L)	28.6 (27.1–30.7)	29.7 (27.9–31.8)^*^a	28.5 (26.6–30.5)	28.3 (26.1–30.9)^*^	28.5 (27–30.9)	29.5 (27.6–30.9)	28.4 (26.2–30.9)	30.3 (28.9–30.3)
A/G	1.6 (1.5–1.8)	1.6 (1.4–1.7)^*^	1.6 (1.5–1.8)	1.6 (1.4–1.8)^**^	1.6 (1.5–1.8)	1.6 (1.5–1.7)^*^	1.6 (1.5–1.7)	1.6 (1.4–1.6)
Uric acid (μmol/L)	318.5 (252–336)	312.5 (275–358)^**^b	274 (241–315)	310 (264–363)^**^b	272 (238–302)	326.5 (225–428)^**^b	281.5 (251–294)	313.5 (264–332)^**^b
TSH (mU/L)	1.95 (1.37–2.69)	2.07 (1.48–2.81)	1.92 (1.36–2.66)	1.92 (1.35–2.69)	1.85 (1.30–2.59)	1.95 (1.35–2.70)	1.77 (1.22–2.45)	1.76 (1.35–2.65)
FT3 (pmol/L)	5.08 (4.60–5.50)	5.00 (4.60–5.50)	5.1 (4.7–5.5)	5.0 (4.7–5.6)^**^	4.9 (4.5–5.3)	5.0 (4.6–5.5)^**^^a^	4.81 (4.45–5.25)	4.90 (4.50–5.45)
FT4 (pmol/L)	11.40 (10.47–12.48)	11.05 (10.05–12.08)^*^a	11.30 (10.30–12.31)	11.19 (10.24–12.20)^*^	11.14 (10.23–12.19)	11.09 (9.90–12.21)^*^	11.06 (10.13–12.05)	10.33 (9.46–11.52)^**^
FT3/FT4	0.44 (0.40–0.49)	0.46 (0.41–0.51)^*^	0.44 (0.40–0.49)	0.45 (0.41–0.51)^**^	0.44 (0.39–0.48)	0.45 (0.40–0.51)^**^b	0.44 (0.40–0.49)	0.47 (0.42–0.53)^**^b

*Data are presented as median (quartiles). Mann-Whitney test and Chi-square test were used to compare differences between PCOS patients and controls*.

* and

** *denote p < 0.05 or p < 0.01 vs. non-PCOS group; a and b denote p < 0.05 or p < 0.01 vs. non-PCOS group after adjusted for BMI*.

### Association Between PRL and Endocrine Metabolic Parameters

To analyze the association between PRL and metabolic parameters in PCOS patients, we grouped the patients according to the quartile PRL levels and analyzed their differences (Table 2). The results showed that in lower PRL quartile, the age, BMI, LH, LH/FSH, TG, TC, LDL-C, AST, ALT, y-GGT, FT3, and FT3/FT4 were higher, while FPG, TSH, and FT4 were lower (*p* < 0.05 or *p* < 0.01).

**Table 2:** Comparison of endocrine and other metabolic parameters in PCOS patients with different PRL levels.

	**PRL level (mIU/L)**				
**Metabolic parameter**	**≤195.89 (*n* = 512)**	**195.90–254.02 (*n* = 515)**	**254.10–337.99 (*n* = 509)**	**>337.99 (*n* = 516)**	***P*–value**
Age(year)	29 (27–32)	29 (27–31)	28 (26–31)	28 (26–31)^*^	0.002
Systolic blood pressure (mmHg)	117 (107–126)	117 (106–125)	117 (106–124)	115 (105–125)	0.226
Diastolic blood pressure mmHg)	73 (68–80)	73 (68.5–80)	73 (69–80)	72 (68–79)	0.605
BMI(kg/m^2^)	23.4 (20.9–25.7)	22.8 (20.4–25.6)	22.3 (20.0–25.6)	22.1 (20.0–24.8)^**^	0.000
PRL (mIU/L)	160.7 (133.6–178.5)	223.9 (209.3–239.0)	289.4 (272.5–308.3)	406.6 (370.5–456.4)^**^	0.000
LH (IU/L)	9.5 (5.65–13.6)	8.1 (5.4–12.3)	8.3 (5.0–13.1)	7.1 (4.9–11.3)^**^	0.000
FSH (IU/L)	6.65 (5.7–7.8)	6.7 (5.7–7.8)	6.7 (5.7–7.8)	6.6 (5.7–7.7)	0.875
LH/FSH	1.4 (0.9–2.0)	1.2 (0.8–1.8)	1.2 (0.8–1.9)	1.1 (0.8–1.7)	< 0.01
E_2_ (pmol/L)	145 (103.0–197.2)	143 (104.5–189.0)	145 (104.0–202.5)	145 (101–195)	0.900
T (nmol/L)	1.8 (1.4–2.3)	1.8 (1.4–2.3)	1.9 (1.4–2.440)	1.8 (1.3–2.3)	0.413
FPG (mmol/L)	5.2 (5.0–5.5)	5.3 (5.1–5.6)	5.3 (5.0–5.5)	5.3 (5.0–5.6)^*^	0.040
TG (mmol/L)	1.3 (0.9–1.9)	1.1 (0.8–1.8)	1.2 (0.8–1.8)	1.1 (0.8–1.7)^*^	0.011
TC (mmol/L)	4.7 (4.2–5.3)	4.6 (4.1–5.2)	4.6 (4.1–5.6)	4.5 (4.0–5.1)^*^	0.002
LDL-C (mmol/L)	2.7 (2.2–3.2)	2.6 (2.2–3.1)	2.6 (2.1–3.1)	2.6 (2.1–3.1)^*^	0.011
HDL-C (mmol/L)	1.3 (1.1–1.6)	1.3 (1.2–1.6)	1.4 (1.2–1.6)	1.4 (1.2–1.6)	0.156
AST (U/L)	19.5 (16–23)	19 (16–24)	19 (16–24)	18 (15–22)^*^	0.002
ALT (U/L)	19 (14–29)	17 (13–29)	18 (12–30)	16 (12–26)^*^	0.001
γ -GGT (U/L)	19 (13.5–33.5)	19 (14–30)	17.5 (12–29)	17 (12–28)^**^	0.000
ALP (U/L)	62 (55–75)	67.5 (57–77)	65 (58.5–80)	62 (56.5–77)	0.069
TBIL (umol/L)	11 (9–13)	11 (9–14)	12.5 (9.4–14.5)	11 (9–13)	0.086
DBIL (umol/L)	3 (2–4)	3 (3–3)	3 (2–4)	3 (2–4)	0.450
TP (g/L)	78.2 (73.5–80.9)	76.45 (74.1–78.6)	77.3 (74.9–79.3)	75.8 (73.2–78.4)	0.605
ALB (g/L)	47.6 (44.8–48.7)	47.4 (46–49.2)	47.1 (43.9–49.5)	47.5 (46.1–49.2)	0.580
GLB (g/L)	29.9 (26–31.6)	28.4 (26.8–30.7)	29.6 (27.8–31.3)	28.3 (26.4–31.7)	0.382
A/G	1.6 (1.4–1.8)	1.6 (1.5–1.8)	1.6 (1.4–1.8)	1.6 (1.5–1.8)	0.225
Uric acid (μmol/L)	332.5 (298–375)	328 (285–375)	316.5 (278–359)	311 (261–380)	0.234
TSH (mU/L)	1.8 (1.3–2.5)	1.8 (1.3–2.7)	2.01 (1.4–2.7)	2.1 (1.5–2.9)^**^	0.000
FT3 (pmol/L)	5.1 (4.7–5.6)	5.1 (4.7–5.6)	5.0 (4.6–5.4)	5.0 (4.6–5.5)^*^	0.024
FT4 (pmol/L)	10.9 (9.9–12.1)	11.1 (10.3–12.3)	11.0 (10.1–12.0)	11.3 (10.0–12.3)^*^	0.019
FT3/FT4	0.5 (0.4–0.5)	0.5 (0.4–0.5)	0.5 (0.4–0.5)	0.4 (0.4–0.5)^*^	0.047

*Data are presented as median (quartiles). Kruskal-Wallis test and Chi-square test were used to compare differences among the four PCOS patient groups*.

* and

** *denote p < 0.05 or p < 0.01 vs. non-PCOS group; a and b denote p < 0.05 or p < 0.01 vs. non-PCOS group after adjusted for BMI*.

### Multivariate Linear Regressions Between PRL and Metabolic Parameters

To further analyze the association between PRL and metabolic parameters, we perform Spearman correlation analysis in the PCOS patients. The results showed that PRL was significantly and positively correlated to FPG (*R* =0.005, *p* < 0.05), TSH (*R* = 0.118, *p* < 0.01), FT4 (*R* = 0.048, *p* < 0.05), and negatively correlated to age (*R* = −0.093, *p* < 0.01), BMI (*R* = −0.115, *p* < 0.01), LH (*R* = −0.100, *p* < 0.01), FH/LSH (*R* = −0.100, *p* < 0.01), TG (*R* = −0.067, *p* < 0.01), TC (*R* = −0.089, *p* < 0.01), LDL-C (*R* = −0.074, *p* < 0.01), AST (*R* = −0.060, *p* < 0.05), ALT (*R* = −0.089, *p* < 0.01), γ -GGT (*R* = −0.110, *p* < 0.01), FT3/FT4 (*R* = −0.070, *p* < 0.01), and uric acid (*R* = −0.049, *p* < 0.05). To adjust the effect of age and BMI, multiple linear regression was used to analyze the relationship between PRL and metabolic parameters. PRL was found to be positively correlated to FPG, TSH, and FT4, and negatively correlated to LH and LH/FSH (Table 3; *p* < 0.05 or *p* < 0.01).

**Table 3:** Multivariate linear regression analysis between PRL and metabolic parameters.

		**Independent**	**Variable**	
**Dependent variable**	**PRL**	**Age**	**BMI**	***R*^**2**^**
LH	−0.109^**^	−0.088^**^	−0.163^**^	0.044
LH/FSH	−0.106^**^	−0.094^**^	−0.106^**^	0.029
FPG	0.052^*^	0.113^**^	0.174^**^	0.047
TG	−0.024	0.083^**^	0.302^**^	0.106
TC	−0.043	0.073^*^	0.063^*^	0.013
LDL-C	−0.027	0.042	0.162^**^	0.031
TSH	0.111^**^	0.001	0.070^*^	0.016
FT4	0.060^*^	−0.006	−0.043	0.006
FT3/FT4	−0.042	0.004	0.138^**^	0.022
AST	−0.013	0.023	0.190^**^	0.038
ALT	−0.013	0.035	0.303^**^	0.097
ALP	−0.024	−0.048^*^	0.233^**^	0.055
γ -GGT	−0.022	0.078^**^	0.268^**^	0.085
Uric acid	−0.017	−0.072^**^	0.406^**^	0.165

*Multivariate linear regression analysis was performed with endocrine and metabolic parameters as dependent variables, PRL, age, and BMI as independent variables*,

* and

** *indicate P < 0.05 and < 0.01, respectively*.

## Discussion

The secretion of PRL is influenced by many factors, such as age and smoking status (39). To exclude the influence of age, we divided the patients into different age groups. The results showed that PRL is significantly lower in PCOS patients than controls in each age group. After adjusted for BMI effect, the difference is still significant. This is consistent with previous study (34). Smoking is shown to reduce serum PRL (40). Glintborg et al. suggested that smoking should be taken into consideration when analyzing the relationship between PRL and metabolic risks (34). However, the percentage of smoking women is only 2.7% for women aged over 15 years in 2019 according to a report from Chinese Center for Disease Control and Prevention. Therefore, smokers were excluded and smoking was not analyzed in our study.

E_2_ can effectively stimulate PRL secretion through pituitary and hypothalamus (41, 42). Dombrowicz et al. suggested that PRL could directly stimulate T production (43). However, we did not find any difference in E_2_ and T levels among the groups. We found significant negative correlation between serum PRL and LH and LH/FSH. Under certain physiological and pathological conditions, high serum PRL above the normal range can directly inhibit the synthesis and release of gonadotropin releasing hormone (GnRH) from the hypothalamus (44) and reduce the secretion of GnRH to the portal vein (45), and high serum PRL also can suppress kisspeptin neurons in the arcuate nucleus (46), resulting in the suppressing the frequency and amplitude of FSH and LH pulses (47, 48). Whether the significant negative relationship between serum PRL and LH or LH/FSH found in our study is an indication that serum PRL in normal range would suppress the secretion of gonadotropin is still not clear. PCOS patients tend to have low quality of life and low mood, which may lead to increased secretion of dopamine and reduced serum PRL levels (18, 49, 50). These may partially explain the negative correlations among PRL, LH and LH/FSH found in this study.

Current studies suggest that serum PRL interacts with glucose metabolism (51). On one side, serum PRL at different levels has different effects on glucose metabolism. Hyperprolactinemia patients were more likely to develop impaired glucose tolerance and insulin resistance (18, 52, 53). These changes can be reduced by using dopamine receptor agonists that reduce serum PRL levels. However, when serum PRL is in normal physiological range, its relationship with glucose metabolism is reversed (22, 27, 54). Low serum PRL may indicate a higher risk of developing metabolic syndrome and type 2 diabetes mellitus (23, 24). On the other side, PRL secretion is affected by blood glucose level. When the blood glucose decreases, the glucose receptor in the hypothalamic neurons will transmit the information to related neurons, stimulating TRH cells and finally regulating PRL secretion (55). However, our study did not find any difference in FPG between PCOS and non-PCOS patients, although serum PRL and FPG is positively correlated in the PCOS patients. These results are inconsistent with previous studies. We speculate that infertile PCOS patients with normal blood glucose may be a special group, whose glucose metabolism is somewhat different from healthy population, although the specific reasons and mechanism are unclear.

Our study found that the BMI of PCOS patients in all age groups is significantly higher than that of non-PCOS patient and the lower serum PRL the higher the BMI. This is consistent with earlier study (56). PRL is produced and released by fat cells and glands in normal human breasts (57), as well as by macrophages in the adipose tissue in response to inflammation and hyperglycemia (6). It promotes the formation of adipose and inhibits the breakdown of lipid in the adipose tissue (7). PRL released from subcutaneous adipose tissue grafted from obese patients is significantly lower than that from non-obese patients (58) and serum PRL of obese children is lower than that of non-obese children (23). Although PRL release is affected by obesity, the mechanism and consequences of decreased PRL release due to obesity are still largely unknown. But, some study showed that PRL is not related to BMI in obese and non-obese patients, and basic serum PRL not related to BMI (59). Our results support the view that obesity decreases PRL release and lows serum PRL. In addition, we showed that even after removing the effects of BMI, the levels of TG, TC and LDL-C in PCOS patients are still significantly higher than in non-PCOS patients and PRL is negatively correlated with TG, TC and LDL-C in the PCOS patients. This is basically consistent with the previous results (60). It can be concluded that lipid metabolism in PCOS patients is abnormal. Since serum PRL is closely related to lipid metabolism, low PRL in physiological range would lead to more serious disorder of lipid metabolism.

Prolactin receptor (PRLR) is highly expressed in the liver, which is the key organ to maintain the homeostasis of metabolism. However, little is known about the role of serum PRL in the liver. Zhang et al. evaluated the expression of PRLR and signaling molecules involved in lipid metabolism in human liver and HepG2 cells, and found that the serum PRL in patients with non-alcoholic fatty liver is lower than that of control (61). They believed that there is a new link between the central nervous system and the liver, and PRL improves steatosis of the liver through PRLR and fat/CD36. In our PCOS patients, the serum PRL level was within normal range and patients with hepatitis and abnormal liver function were excluded The analysis showed that PRL is negatively correlated with AST, ALT, γ –GGT, and ALP, suggesting that lower serum PRL may damage liver cells. Even after removing the effects of BMI, the levels of AST, ALT γ -GGT, ALP, and uric acid are significantly higher in PCOS and in non-PCOS patients. Moreover, our results showed that the lower the serum PRL, the higher the serum TG, TC, and LDL. Therefore, it is necessary to further study whether there are other mechanisms related to hepatic microcirculatory dysfunction caused by lipid metabolism disorder under low PRL condition. Since PRL regulates enzymes and transporters related to glucose and lipids in other target organs, future studies should be performed to investigate the pathophysiological effects on liver tissue.

Thyroid gland and gonad are controlled by pituitary gland. The secretion of PRL and TSH is controlled by the neuroendocrine regulation mechanism of triiodothyronine (T3), thyroxine (T4), thyrotropin releasing hormone (TRH), and dopamine. TRH can not only promote the release of TSH, but also promote the release of PRL (62, 63). TSH is confirmed to be closely associated with metabolic syndrome (64). On the one hand, thyroid hormone acts on tissues and organs such as the liver and participate in the synthesis and decomposition of TG and TC through multiple pathways (65). On the other hand, TSH receptor is also expressed in tissues other than thyroid, such as liver and adipocytes, where TSH interacts with the receptors to play a number of biological functions (61, 66, 67). For example, TSH can promote the synthesis of cholesterol in hepatocytes in a dose-time-dependent manner after binding to the receptor on the membrane of hepatocytes, and can also reduce the activity of cholesterol 7 α-hydroxylase to participate in cholesterol transformation (66). Human adipocytes and preadipocytes also have TSH receptors. TSH can induce preadipocytes to differentiate into adipocytes by binding to the receptors, thus promoting adipogenesis (68). TSH can also facilitate the decomposition of lipid by phosphorylating lipid body coating proteins and hormone- sensitive lipase (69). Most of the previous studies showed that the level of TSH is positively correlated with TG, TC and LDL-C in either hypothyroidism, subclinical hypothyroidism or normal population (70–72). We found that PRL is positively related to TSH. However, in low PRL patients, TSH is lower but TG, TC, and LDL-C are higher, which is contrary to previous studies. This difference might be attributed to the differences in metabolic mechanisms between the PCOS infertile patients and other populations. Well-designed prospective studies are needed to have a better understand of involvement and pathophysiological significance of PRL in lipid metabolism via TSH, and would help elucidate the differences.

In addition, in healthy young men, the less favorable body compositions (or higher fat and lower muscle mass, with higher leptin concentration) are shown to be correlated with insulin resistance and FT3 levels in normal thyroid function (73) and in healthy middle-aged men and women with normal thyroid function, TSH, FT3, and FT3/FT4 are positively correlated with BMI, waist circumference and components of metabolic syndrome (such as TG, SBP, DBP, and FPG), and negatively correlated with FT4 (74). We found that SBP and DBP in different age groups and FT3/FT4 in patients older than 31 years are significantly higher in PCOS and non-PCOS patients, while FT4 is significantly lower. In our cohort, PRL is positively related to FT4, and negatively to FT3/FT4 and FT3 (although not statistically significant) and to TG, TC, and LDL-C. These results are consistent with previous studies. Therefore, we concluded that the low PRL in infertile PCOS patients is an effective marker of poor metabolic spectrum and higher cardiovascular risk.

As a retrospective analysis, there were a number of limitations in this study. For instance, PRL secretion occurs as pulse and is better measured in repeated samples from venous catheter to ensure the accuracy. In addition, the ratio of waist to hip is closely related to metabolism, but the relevant data was not available. For thyroid function assessment, information on thyroid-associated antibodies was absent. Patients with fatty liver were unable to be excluded due to the lack of ultrasound examination. Furthermore, only fasting blood glucose content, no fasting insulin data and glucose tolerance data were available.

In conclusion, our study suggested that low serum PRL may be an important cause of metabolic risk in infertile PCOS patients.

## Data Availability Statement

The datasets used and/or analyzed during the current study are available from the corresponding author on reasonable request.

## Ethics Statement

The studies involving human participants were reviewed and approved by Ethics Committee of the First Affiliated Hospital, Wenzhou Medical University, China. The patients/participants provided their written informed consent to participate in this study.

## Author Contributions

HY and JD: project conceptualization, investigation, and data analysis. JD, JP, and RY: data collection, analysis, and methodology development. RY and YT: data collection, analysis, and methodology development. ZC and XD: investigation and methodology development. XD: project conceptualization and manuscript writing. HY, JD, and XD: project conceptualization, investigation, and manuscript writing.

## Conflict of Interest

The authors declare that the research was conducted in the absence of any commercial or financial relationships that could be construed as a potential conflict of interest.

## Abbreviations

PRL: Prolactin
PCOS: Polycystic ovary syndrome
IVE-ET: *In vitro* fertilization and embryo transfer
FPG: Fasting plasma glucose
BMI: Body mass index
TSH: Thyroid stimulating hormone
FT4: Free thyroxine
FT3: Free triiodothyronine
LH: Luteinizing hormone
FSH: Follicle stimulating hormone
LDL-C: Low density lipoprotein cholesterol
HDL-C: High density lipoprotein cholesterol
TC: Total cholesterol
TG: Triglyceride
AST: Aspartate aminotransferase
ALT: Alanine aminotransferase
Y-GGT: Glutaminyl transferasel
ALP: Alkaline phosphatase
T: Testosterone
E2: Estradiol
TBIL: Total bilirubin
DBil: Direct bilirubin
TP: Total protein
ALB: Albumin
SBP: systolic blood pressure
DBP: diastolic blood pressure.
