# Supplementary material for: The Association Between Prolactin and Metabolic Parameters in PCOS Women: A Retrospective Analysis
Source: Front Endocrinol (Lausanne). 2020 May 12;11:263. doi: 10.3389/fendo.2020.00263 (PMC7235367; doi:10.3389/fendo.2020.00263)
Supplement: Supplementary file 1 [file Table_1.docx]

Supplementary Table 1. Clinical data of PCOS and non-PCOS patients

| Variables | Non-PCOS (n=9696) | PCOS (n=2052) | P-value |
| --- | --- | --- | --- |
| Age (year) | 31(28-34) | 29(27-31) ** | 0.000 |
| Systolic pressure (mmHg) | 112（104-121） | 117（106-125）** | 0.000 |
| Diastolic pressure (mmHg) | 70(66-77) | 73(68-80) ** | 0.000 |
| BMI（kg/m^2^） | 21.09(19.53-23.11) | 22.66(20.31-25.39) ** | 0.000 |
| PRL（mIu/L） | 278.82(210.12-365.19) | 254.02(195.89-337.99) ** | 0.000 |
| LH（IU/L） | 4.38(3.30-5.78) | 8.17(5.14-12.64) ** | 0.000 |
| FSH（IU/L） | 7.60(6.45-8.99) | 6.67(5.67-7.78) ** | 0.000 |
| E_2_（pmol/L） | 140(100-192) | 145(103-197.10) * | 0.005 |
| T（nmol/L） | 1.24(0.96-1.59) | 1.81(1.39-2.32) ** | 0.000 |
| FPG (mmol/L） | 5.4(5.1-6.1) | 5.3(5.0-5.6) | 0.862 |
| TG (mmol/L） | 0.88(0.64-1.25) | 1.17(0.80-1.80) ** | 0.000 |
| TC (mmol/L） | 4.38(3.89-4.90) | 4.60(4.10-5.19) ** | 0.000 |
| LDL-C (mmol/L） | 2.40(2.03-2.83) | 2.60(2.17-3.11) ** | 0.000 |
| HDL-C (mmol/L） | 1.44(1.24-1.65) | 1.33(1.14-1.58) ** | 0.000 |
| AST (U/L) | 18.5（15-24） | 20（17-26） | 0.000 |
| ALT (U/L) | 13（10-19） | 17（13-29） | 0.000 |
| Ƴ-GGT (U/L) | 15（11-22.5） | 18（13-31） | 0.000 |
| ALP (U/L) | 58（50-68.5） | 65（57-77） | 0.000 |
| TBIL (umol/L) | 12（10-15） | 11（9-14） | 0.000 |
| DBIL (umol/L) | 3（2-4） | 3（2-3.5） | 0.022 |
| TP (g/L) | 76（73.7-78.8） | 76.4（74.2-79.2） | 0.000 |
| ALB (g/L) | 47.1（45.2-48.8） | 47.4（45.5-49.2） | 0.048 |
| GLB (g/L) | 28.5（26.7-30.8） | 29（27-31） | 0.005 |
| A/G | 1.6（1.5-1.8） | 1.6（1.5-1.8） | 0.000 |
| Uric acid (µmol/L) | 294.5（255.5-336.5） | 323（279-374） | 0.000 |
| TSH (mU/L) | 1.87(1.32-2.61) | 1.93(1.35-2.70) | 0.008 |
| FT3 (pmol/L) | 4.92(4.53-5.40) | 5.00(4.63-5.50) | 0.000 |
| FT4 (pmol/L) | 11.23(10.30-12.27) | 11.09(10.06-12.18) | 0.000 |

Data are presented as median (quartiles). Mann-Whitney test and Chi square test were used to compare differences between PCOS patients and controls.* and ** denote *p* < 0.05 or *p* < 0.01 vs non-PCOS group
